# Maternal diet quality and circulating extracellular vesicle and particle miRNA during pregnancy

**DOI:** 10.1007/s00394-025-03589-x

**Published:** 2025-02-01

**Authors:** Meghan E. Muse, Yuting Wang, Diane Gilbert-Diamond, David A. Armstrong, Anne G. Hoen, Megan E. Romano, Jiang Gui, Thomas J. Palys, Frederick W. Kolling, Brock C. Christensen, Margaret R. Karagas, Caitlin G. Howe

**Affiliations:** 1 Department of Epidemiology, Geisel School of Medicine at Dartmouth, 1 Medical Center Dr, Lebanon, NH USA; 2 Research Service, V.A. Medical Center, White River Junction, VT USA; 3 Department of Dermatology, Dartmouth Health, Lebanon, NH USA; 4 Department of Biomedical Data Science, Geisel School of Medicine at Dartmouth, Lebanon, NH USA; 5 Dartmouth Cancer Center, Geisel School of Medicine at Dartmouth, Lebanon, NH USA; 6 Department of Molecular and Systems Biology, Geisel School of Medicine at Dartmouth, Lebanon, NH USA

**Keywords:** Pregnancy, Diet, Alternative Healthy Eating Index 2010, Plasma, Extracellular vesicles, miRNA

## Abstract

**Purpose:**

During pregnancy, extracellular vesicle and particle microRNAs (EVP miRNA) in maternal circulation have the capacity to cross the placenta and facilitate maternal-fetal communication. Both dysregulation of circulating EVP miRNA during pregnancy and maternal diet quality have been previously associated with pregnancy complications and adverse birth outcomes. However, little is known about how maternal diet influences circulating EVP miRNA during pregnancy. This study assesses associations between maternal diet quality, as measured by the Alternative Healthy Eating Index (2010; AHEI-2010), and EVP miRNA levels in maternal circulation during pregnancy.

**Methods:**

In a pilot study of 53 pregnant participants in the New Hampshire Birth Cohort Study, maternal diet quality was assessed using AHEI-2010 and plasma (mean gestational age at blood collection: 28.8 weeks) EVP miRNA were profiled using the NanoString nCounter platform which interrogates 798 miRNA transcripts.

**Results:**

In covariate-adjusted models, the AHEI-2010 adherence score was negatively associated (*P* < 0.05) with the number of unique miRNA transcripts detectable in each sample. In post hoc analyses, greater consumption of red and processed meats was positively associated with levels of 7 miRNA (*Q* < 0.05), including hsa-miR-512-5p (*P*_*Bonf*_ < 0.01), a member of the placenta-specific chromosome 19 miRNA cluster.

**Conclusion:**

We identified associations between the consumption of red and processed meat and levels of circulating select EVP miRNA during pregnancy, including placenta-specific miRNA and miRNA with target genes overrepresented in pathways involved in placental development. Additional research is needed to assess whether alterations in maternal circulating EVP miRNA may mediate maternal diet quality’s impacts on pregnancy and birth outcomes.

**Supplementary Information:**

The online version contains supplementary material available at 10.1007/s00394-025-03589-x.

## Introduction

MicroRNAs (miRNA) are short, non-coding RNA transcripts which regulate gene expression post-transcriptionally. In addition to regulating gene expression in their cell of origin, miRNA can be loaded into extracellular vesicles and other extracellular particles (EVPs) for transport to new target cells, either proximally or in distal tissues [1]. EVPs found in circulation originate from a range of cell and tissue types, and during pregnancy a large proportion are of placental origin with increasing levels across gestation [2, 3]. EVPs can cross the placenta, mediating communication between the maternal and fetal compartments during pregnancy [4].

Dysregulation of maternal circulating EVP miRNA has been associated with adverse pregnancy and birth outcomes, including hypertensive disorders of pregnancy [5–7], gestational diabetes [8], shorter gestation [9], and low birth weight [9]. Although prior studies indicate that maternal circulating EVP miRNA may be altered in response to adverse maternal exposures, such as toxic metals [10, 11] and psychosocial stress [12], little is known about their potential sensitivity to health-promoting behaviors and exposures.

Prior work has demonstrated the importance of maternal diet quality during pregnancy for both maternal and child health. For example, higher diet quality during pregnancy has been associated with better child health outcomes, including improved child appetitive traits [13], reduced adiposity [14–17], and better cognitive outcomes [18, 19]. These prior studies largely measured diet quality using the Alternative Healthy Eating Index 2010 (AHEI-2010) which scores the consumption of foods previously linked with either a higher or lower likelihood of developing chronic disease [20]. AHEI-2010 adherence scores increase with higher consumption of fruits, vegetables, whole grains, nuts and legumes, long chain fatty acids (EPA + DHA), and polyunsaturated fats and lower consumption of sugar sweetened beverages, red and processed meats, trans fats, and sodium. In pregnancy, higher diet quality, as measured by AHEI-2010, has been linked to a decreased risk of pregnancy complications [21, 22] as well as a decreased risk of infants being born small for gestational age [23, 24].

Although dietary interventions have previously been shown to alter levels of circulating EVP miRNA in non-pregnant individuals [25, 26], there is a lack of research evaluating the association between maternal dietary patterns during pregnancy and levels of circulating EVP miRNA during pregnancy. Here, we assess the association between maternal diet quality, as measured by AHEI-2010, and circulating EVP miRNA measured in maternal plasma during pregnancy in 54 participants in the New Hampshire Birth Cohort Study.

## Methods

### Study population and diet quality assessment

As previously described [11], 54 mother-infant pairs enrolled between 2014 and 2016 were selected from the New Hampshire Birth Cohort Study (NHBCS) for EVP miRNA profiling. Selection criteria included having provided a blood sample during pregnancy and availability of covariates, such as maternal age, pre-pregnancy BMI, and educational attainment. Because an aim of the initial pilot study was metal impacts on human milk-derived EVP miRNA [11], additional selection criteria included donation of bilateral milk samples at approximately 6 weeks postpartum, exclusive breast feeding for a minimum of 6 months, and collection of maternal toenail clippings during pregnancy and postpartum for metal exposure assessment. All study protocols were approved by the Institutional Review Board at Dartmouth College and all participants provided written informed consent at the time of enrollment.

During the prenatal study visit at approximately 24–28 weeks gestation, participants completed a validated food frequency questionnaire (FFQ) [27] reporting their food consumption over the past 12 months, including the preconception period. FFQ responses were used to calculate total daily caloric intake and adherence scores for the Alternative Healthy Eating Index-2010 (AHEI-2010) [20] based on food composition data from the US Department of Agriculture food composition table [28]. Briefly, increased consumption of vegetables, fruits, whole grains, nuts, legumes, and soy, EPA and DHA, and polyunsaturated fats are positively scored from 0 to 10 while the decreased consumption of sugar sweetened beverages, red and processed meats, trans fats, and sodium are positively scored from 0 to 10 and moderate alcohol consumption is scored 0–10 with a score of 0 reflecting high consumption (2.5 or more drinks per day), 2.5 reflecting no consumption, and 10 reflecting moderate consumption. The final range for overall AHEI-2010 score is 0-110.

### Blood collection and plasma EVP miRNA extraction and profiling

The collection and processing of maternal blood samples during pregnancy has been described in detail previously [29]. Briefly, maternal peripheral blood samples were collected in K2EDTA tubes at approximately 24–28 weeks gestation, fractioned by centrifugation within 24 h of collection, and plasma aliquots were stored at -80 °C. Using the Qiagen Plasma/Serum exoRNeasy Kit (QIAGEN), EVP miRNA were extracted from 500 µl of plasma. Extraction efficacy was monitored using a synthetic miRNA spike-in from *Oryza sativa* (osa-miR-414; 200 pM) and eluted miRNA were purified using Amicon Ultra Centrifugal Filters (Millipore, Billerica, MA, USA) and concentrated using a speed vacuum concentrator.

Levels of 798 miRNA were quantified using the NanoString nCounter Expression Assay Human v3 (Nanostring Technologies, Inc. Seattle, WA, USA) on the nCounter Analysis System. These data are publicly available under the Gene Expression Omnibus (GEO) accession code GSE223273.

### Data processing

Data processing and statistical analyses were conducted in R (version 4.1.0). As previously described [29], raw miRNA counts were normalized to sample-specific positive controls using the package *NanoStringNorm* (version 1.2.1.1) [30]. For each sample, the EVP miRNA limit of detection was defined as the mean + 1.5 standard deviations of the sample-specific negative controls. Previously defined summary measures of EVP miRNA composition [29] were calculated for each sample as follows:

Total miRNA counts were calculated as the sum of the normalized counts for the 798 miRNA measured on the NanoString platform. Sample richness was calculated as the absolute count (bound between 0 and 798) of unique miRNA transcripts with levels above the sample-specific detection threshold. Sample evenness (bound between 0 and 1) was calculated from the relative abundance (*p*_*i*_) of each miRNA (*i*) and the sample richness (*s*) such that

$$\:Evenness=\frac{-{\sum\:}_{i=1}^{s}{p}_{i}{ln}\left({p}_{i}\right)}{\text{ln}\left(s\right)}$$

### Primary statistical analysis

Associations between AHEI-2010 adherence scores and measures of EVP miRNA composition were assessed using robust linear regression with the *MASS* package (version 7.3–54) in R [31]. Due to its non-normal distribution across samples, total miRNA counts were log_2_-normalized for all statistical analyses. Model covariates were identified using a directed acyclic graph (Figure S1), and final models were adjusted for education attainment (college graduate vs. non-college graduate), average hours of vigorous physical activity per week at the time of blood collection (continuous), and time of day of blood collection (12PM– 4PM vs. 7AM– 12PM). Due to one participant not reporting vigorous physical activity, the final sample size for this analysis was 53 participants. Additional sensitivity analyses were conducted for statistically significant (*P* < 0.05) findings (1) restricting to never smokers (*n* = 50), (2) excluding participants diagnosed with gestational diabetes in this pregnancy (*n* = 2), (3) excluding participants diagnosed with gestational hypertension during this pregnancy (*n* = 4), and (4) additionally adjusting models for pre-pregnancy BMI.

### Secondary analysis: AHEI-Component scores and EVP miRNA composition

For miRNA composition measures that were statistically significantly (*P* < 0.05) associated with AHEI-2010 adherence, we conducted subsequent analyses assessing the AHEI-2010 component scores as predictors. These component scores included vegetables; fruits; whole grains; sugar sweetened beverages; nuts, legumes, and soy; long chain fatty acids (EPA + DHA); polyunsaturated fats; red and processed meats; trans fats; and sodium. As with the primary analyses, these secondary analyses were conducted using robust linear regression with the *MASS* package. These models were adjusted for education attainment (college graduate vs. non-college graduate), average hours of vigorous physical activity per week at the time of blood collection (continuous), and time of day of blood collection (12PM– 4PM vs. 7AM– 12PM), and total daily caloric intake (continuous).

### Post Hoc analyses: diet quality and individual EVP miRNA

For AHEI-2010 adherence and component scores that were statistically significantly associated (*P* < 0.05) with miRNA richness or evenness, post hoc analyses were conducted for the individual miRNA associated with richness and evenness scores in plasma EVP miRNA samples from this study, respectively, as previously reported [29]. Briefly, EVP miRNA that were statistically significantly associated (P_Bonferonni_ < 0.05) with richness or evenness in robust linear models were considered richness- or evenness-associated, respectively, and tested in post hoc analyses.

MiRNA detectable in 20–60% of plasma samples were modeled as binary variables (detectable vs. not detectable) using robust logistic regression with the *MASS* package. MiRNA detectable in more than 60% of samples were modeled as continuous log_2_-transformed counts using robust linear regression models with the *MASS* package. MiRNA detectable in less than 20% of samples were excluded from analysis. All analyses were adjusted for the same covariates that were included in primary analyses. FDR correction was applied to all analyses, and dietary scores were considered statistically significantly associated with a given miRNA at *Q* < 0.05. Additional sensitivity analyses were conducted for statistically significant (*Q* < 0.05) findings: (1) restricting to never smokers (*n* = 50), (2) excluding participants diagnosed with gestational diabetes in this pregnancy (*n* = 2), (3) excluding participants diagnosed with gestational hypertension during this pregnancy (*n* = 4), (4) additionally adjusting for pre-pregnancy BMI, (5) restricting to participants who reported taking a multivitamin (*n* = 51), and (6) restricting to participants who reported not taking medication for morning sickness (*n* = 45).

High confidence predicted target genes of AHEI-2010 and component score-associated miRNA (*Q* < 0.05) were identified using mirDIP (version 5.2) [32]. Enrichment analyses of identified target genes were conducted using *enrichR* (version 3.2) [33] to assess overrepresentation in PANTHER (2016) pathways [34].

## Results

### Participant demographics

Of the 53 participants included in this analysis, the mean daily caloric intake was 2,039.4 kilocalories (kcal) per day (SD: 622.3 kcal/day) the mean AHEI-2010 adherence score was 64.7 with a standard deviation of 10.3 (Table 1). Nuts, legumes, and soy had the highest median AHEI-2010 component score at 9.4 (IQR: 5.0, 10.0; Table S1) which to equates to eating, on average, 0.94 servings of nuts, legumes, or soy per day. The median component score for red and processed meat consumption was 5.73 (IQR: 3.73, 7.07) equating to an average of 0.64 servings per day. Polyunsaturated fat consumption had a median component score of 6.94 (IQR: 5.94: 7.92), which equates to participants getting, on average, 6.94% of their daily caloric intake form polyunsaturated fats. The mean pre-pregnancy BMI was 23.9 kg/m^2^, and the mean gestational age at blood collection was 28.8 weeks gestation (Table 1).

**Table 1:** Participant and second-trimester plasma characteristics (*n* = 53)

Characteristic	Mean (SD), Median [IQR], or *n* (%)
**Age** (yrs)	32.5 (4.0)
**Education Attainment**	
*Some College or Less*	6 (11.3)
*College Graduate or More*	47 (88.7)
**Pre-Pregnancy BMI** (kg/m^2^)	23.9 (5.0)
**AHEI-2010 Score** (0–110)	64.7 (10.4)
**Total Caloric Intake** (kcal/day)	2039.4 (622.3)
**Vigorous Physical Activity** (hrs/wk)	0.0 [0.0, 1.0]
**Infant Sex**	
*Female*	30 (56.6)
*Male*	23 (43.4)
**Gestational Age at Blood Collection** (wks)	28.8 (2.8)
**Time of Day of Blood Collection**	
*7AM – 12PM*	27 (50.9)
*12PM – 4PM*	26 (49.1)
**Total EVP miRNA Counts**	18756.6 [16204.8, 22978.8]
**Sample Richness**	191.7 (73.4)
**Sample Evenness** (0–1)	0.9 (0.1)

### Overall AHEI-2010 findings

In covariate-adjusted models, the AHEI-2010 adherence score was negatively associated with sample richness such that every one-point increase in AHEI-2010 was associated with the detection of 3.0 fewer unique miRNA transcripts in plasma EVPs (95% CI: -4.9 -1.2; *P* = 0.003, Table 2). No statistically significant associations (*P* < 0.05) were observed between AHEI-2010 score and total counts of plasma EVP miRNA nor plasma evenness scores.

**Table 2:** Covariate adjusted, linear associations between AHEI-2010 and miRNA composition measures

	Effect Size^a^ (95% CI)	*P* Value
Total miRNA Counts^b^	-0.002 (-0.012, 0.008)	0.712
Sample Richness	-3.048 (-4.928, -1.168)	0.003
Sample Evenness	-0.001 (-0.002, 0.001)	0.502

^a^ Each miRNA composition measure was modeled separately as the dependent variable using robust linear regression. All models were adjusted for educational attainment (binary), vigorous physical activity (hours/week), and time of day at sample collection (binary)

^b^ Effect sizes and 95% confidence intervals reflect the difference in log_2_-transformed total miRNA counts for a 1-unit increase in AHEI-2010 score

**Fig. 1:**
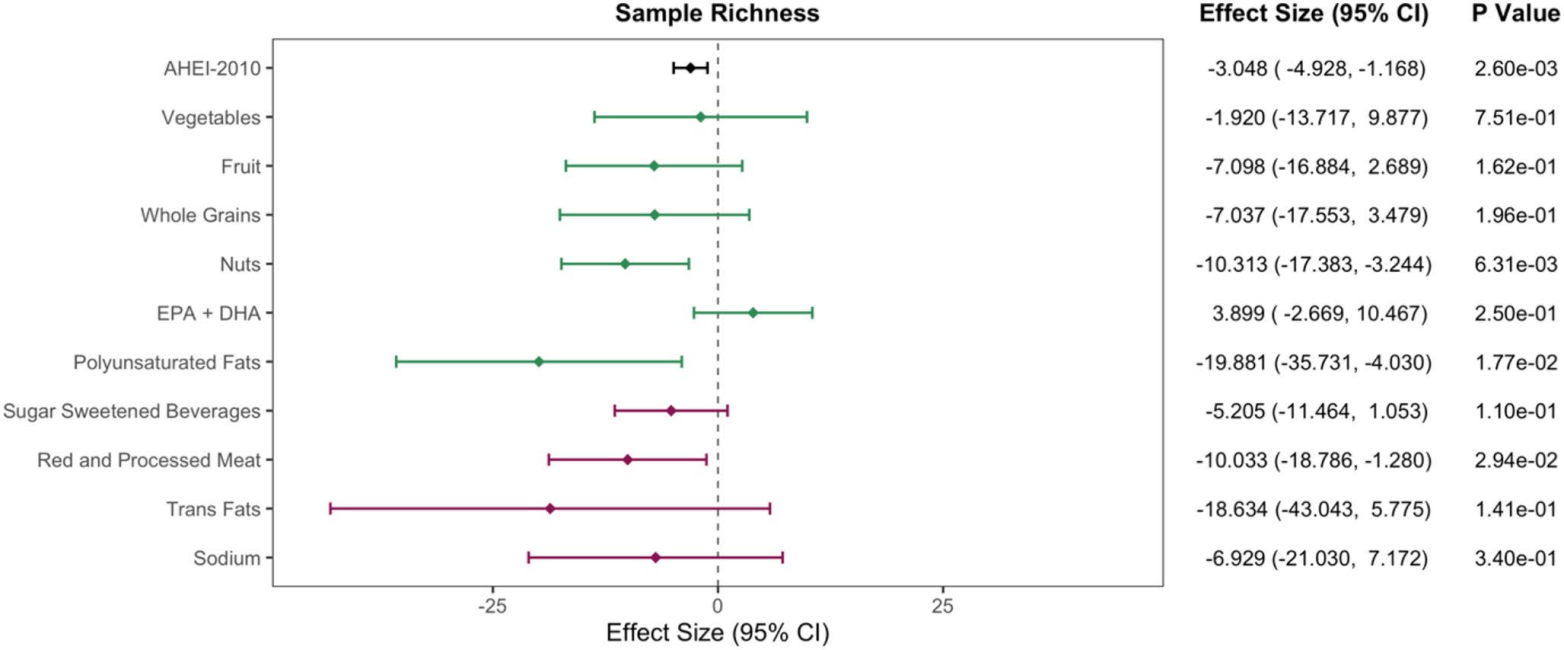
Covariate-adjusted associations between the overall AHEI-2010 score and each of its component scores with sample richness. Sample richness reflects the number of unique miRNA transcripts detected in a sample. Each component score was modeled separately as the dependent variable using linear regression. All models were adjusted for educational attainment (binary), vigorous physical activity (hrs/week) and time of day at blood collection (binary). Additionally, models for component scores were adjusted for total caloric intake. Results in green reflect positively scored dietary components and results in magenta reflect negatively scored dietary components of AHEI-2010

When evaluating the different subcomponents comprising the AHEI-2010 in relation to sample richness, the component scores for nuts, legumes, and soy (-10.3; 95%CI: -17.4, -3.2; *P* = 0.006) and polyunsaturated fats (-19.9; 95% CI: -35.7, -4.0; *P* = 0.018), as well as the reverse-coded component score for red and processed meat (-10.0, 95% CI: -18.8, -1.3; *P* = 0.029), were all negatively associated (*P* < 0.05) with sample richness (Fig. 1). These associations were consistent in sensitivity analyses (1) restricting to never smokers (*n* = 50), (2) excluding participants diagnosed with gestational diabetes in this pregnancy (*n* = 2), (3) excluding participants diagnosed with gestational hypertension during this pregnancy (*n* = 4), (4) additionally adjusting for pre-pregnancy BMI, (5) restricting to participants who reported taking a multivitamin (*n* = 51), and (6) restricting to participants who reported not taking medication for morning sickness (*n* = 45; TableS2).

### AHEI-2010 and richness-associated miRNA

**Fig. 2:**
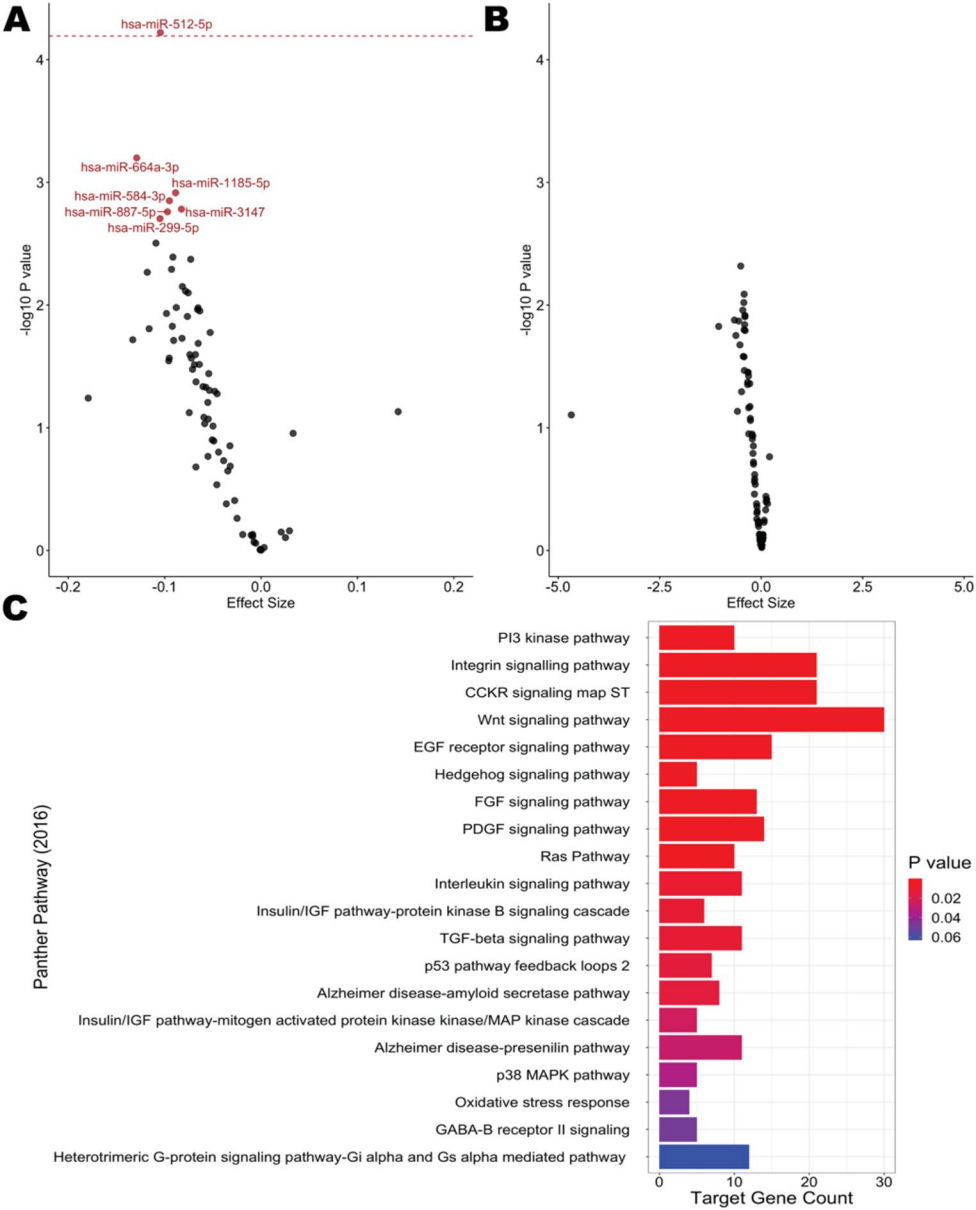
(**A**)Volcano plot of covariate-adjusted associations between the AHEI-2010 component score for red and processed meat consumption and richness-associated miRNA detectable in more than 60% of samples. MiRNA counts were log_2_-transformed and modeled using robust linear regression adjusting for maternal educational attainment (binary), vigorous physical activity (hrs/wk), time of day at blood collection (binary), and total caloric intake (kcal/day). Statistically significant associations after FDR correction (Q < 0.05) are colored in red and labeled. The red dashed line reflects a Bonferroni-adjusted significance threshold of *P*_*Bonf*_ < 0.01. (**B**) Volcano plot of covariate-adjusted associations between the AHEI-2010 component score for red and processed meat consumption and richness-associated miRNAs detectable in 20–60% of samples. EVP miRNA treated as binary variables (detectable vs. not detectable) were modeled using robust logistic regression. Models were adjusted for maternal educational attainment (binary), vigorous physical activity (hrs/wk), time of day at blood collection (binary), and total caloric intake (kcal/day). (**C**) Results from enrichment analyses conducted using EnrichR for the 1,154 high confidence target genes (identified by mirDIP) for the 7 red and processed meat consumption-associated miRNA (*Q* < 0.05)

After FDR correction, no statistically significant associations (*Q* < 0.05) were observed between the overall AHEI-2010 score or component scores for nuts, legumes, and soy or polyunsaturated fats in relation to the individual EVP miRNA associated with sample richness (Table S3). However, the AHEI-2010 component score for red and processed meat consumption was negatively associated (*Q* < 0.05) with the levels of 7 miRNA (hsa-miR-1185-5p, hsa-miR-299-5p, hsa-miR-3147, hsa-miR-512-5p, hsa-miR-584-3p, hsa-miR-664a-3p, hsa-miR-887-5p) in plasma EVPs (Fig. 2; Table S3). Given the reverse-coding of the component score for red and processed meat consumption, this indicates a positive association between servings of red and processed meat per day and the levels of these EVP miRNA. After a more conservative Bonferroni correction, every one-point increase in the reverse-coded component score for red and processed meat consumption was associated with a 0.105 log_2_-fold lower hsa-miR-512-5p count (95% CI: -0.151, -0.058; *P*_*Bonf*_ = 0.0094). These associations were consistent in sensitivity analyses (1) restricting to never smokers (*n* = 50), (2) excluding participants diagnosed with gestational diabetes in this pregnancy (*n* = 2), (3) excluding participants diagnosed with gestational hypertension during this pregnancy (*n* = 4), (4) additionally adjusting for pre-pregnancy BMI, (5) restricting to participants who reported taking a multivitamin (*n* = 51), and (6) restricting to participants who reported not taking medication for morning sickness (*n* = 45; Table S4).

For the 7 red and processed meat-associated EVP miRNA, we identified 1,154 unique high confidence predicted target genes annotated in mirDIP (version 5.2). These 1,154 predicted target mRNA transcripts are overrepresented in 19 PANTHER 2016 pathways (*P*_*BH*_ < 0.05, Fig. 2B) including the PI3 kinase, integrin signaling, CCKR signaling, and Wnt signaling pathways.

## Discussion

In this study, we assessed the association between maternal diet quality and the miRNA composition of circulating EVPs from 53 maternal plasma samples collected during pregnancy. Increased diet quality, as measured by the Alternative Healthy Eating Index 2010 (AHEI-2010) was associated with fewer unique miRNA transcripts being detected in circulating EVPs. In post hoc analyses evaluating the individual components of the AHEI-2010 score, we found that consumption of nuts, legumes, and soy and polyunsaturated fats were associated with fewer unique miRNA in maternal circulating EVPs, while the opposite was true for the consumption of red and processed meats.

In additional post hoc analyses, we found that higher consumption of red and processed meats was associated with higher levels of seven individual miRNA after FDR correction. One of these miRNA (hsa-miR-512-5p) remained statistically significantly associated with red and processed meat consumption after a more conservative Bonferroni correction. Prior in vitro studies have reported that the expression of hsa-miR-512-5p is sensitive to selenium, for which red meat is a major dietary source [35]. Importantly, hsa-miR-512-5p is a member of the placental-specific Chromosome 19 miRNA Cluster (C19MC) and is exclusively detected in plasma during pregnancy [36, 37]. Previously, lower placental and plasma EVP levels of C19MC miRNA were associated with an increased risk of pregnancy complications including preeclampsia, gestational hypertension, and fetal growth restriction, although this has not been observed for hsa-miR-512-5p specifically [37, 38].

Higher daily consumption of red and processed meats was also associated with higher levels of hsa-miR-299-5p, a member of another placental-specific cluster (Chromosome 14 miRNA cluster C14MC). Lower circulating levels of C14MC miRNA has been associated with increased risk for preterm labor [39] and higher levels of hsa-miR-299-5p in circulating EVPs have previously been linked to longer gestational duration in male infants [9]. The upregulation of hsa-miR-299 in placental tissue has also been mechanistically linked to preeclampsia via suppression of trophoblast invasion and migration [40]. Given that C19MC and C14MC miRNA are almost exclusively expressed in the placenta, these results suggest that placental expression of hsa-mir-512-5p and hsa-miR-299-5p may be sensitive to maternal red and processed meat consumption during pregnancy, which could have potential implications for pregnancy complications and birth outcomes. The consumption of red and processed meats has also been linked to increased risk of gestational hypertension and preeclampsia [41], therefore, these findings may provide preliminary evidence of a possible biological mechanism through which red and processed meat consumption might impact gestational hypertension and preeclampsia risk, however given our small sample size, additional research is needed to further explore this possibility.

Predicted target genes of the 7 EVP miRNA associated with red and processed meat consumption were enriched in the CCKR signaling, Wnt signaling, and integrin signaling pathways. CCKR signaling is well-documented to play an important role in digestion, and prior studies in mice have shown that the CCKR and Wnt signaling pathways are altered in response to dysregulated miRNA following the consumption of high fat meals [42, 43]. During pregnancy, the Wnt signaling pathway also plays an important role in trophoblast development and differentiation [44, 45], and prior work has linked the dysregulation of this pathway to preeclampsia and gestational diabetes [46, 47]. Additionally, the integrin signaling pathway is involved in the regulation of glucose and lipid metabolism in mice [48, 49]. A recent study of lean red meat consumption in non-pregnant women identified a positive association between lean red meat consumption and circulating levels of miR-15b-5p, a miRNA not assessed in our study, as well as an inverse association between miR-15b-5p and plasma insulin levels [50]. Thus, prior evidence supports that the pathways that we found to be related to red and processed meat consumption in the current study are sensitive to diet. Additional work is needed to assess whether biochemical profiles related to these pathways are perturbed with consumption of red and processed meat during pregnancy and whether such perturbation is mediated by levels of circulating EVP miRNA.

This study was strengthened by the collection of dietary data using a validated food frequency questionnaire for exposure assessment and robust covariate adjustment. Due to the small sample size, primary analyses assessed relationships between overall maternal diet quality and three measures of EVP miRNA composition in maternal circulation during pregnancy. Although in post hoc analyses we also identified specific dietary components that may influence EVP miRNA composition, as well as several individual miRNA that may be sensitive to red and processed meat consumption, additional work is needed to assess associations between diet quality and specific dietary components with individual EVP miRNA as we may have been underpowered to fully explore these relationships. Additionally, while we selected AHEI-2010 as our measure of overall maternal diet quality given prior associations reported between AHEI-2010 and birth outcomes, including in the same cohort [24], further work is needed to understand whether other measures of maternal diet influence circulating EVP miRNA during pregnancy. Overall, findings from this study suggest that maternal diet quality, and more specifically the consumption of red and processed meat, may alter circulating EVP miRNAs during pregnancy, including placenta-specific miRNA that are dysregulated in pregnancy complications and adverse birth outcomes and play a regulatory role in pathways involved in insulin secretion and placental development. Further work is needed to better understand the downstream molecular implications of these alterations and whether they influence maternal or child health outcomes.

## Electronic supplementary material

Below is the link to the electronic supplementary material.

Supplementary Material 1

Supplementary Material 2
