# Supplementary material for: Maternal diet quality and circulating extracellular vesicle and particle miRNA during pregnancy
Source: Eur J Nutr. 2025 Feb 1;64(2):75. doi: 10.1007/s00394-025-03589-x (PMC11787256; doi:10.1007/s00394-025-03589-x)
Supplement: Supplementary file 1 — Supplementary Material 1 [file 394_2025_3589_MOESM1_ESM.docx]

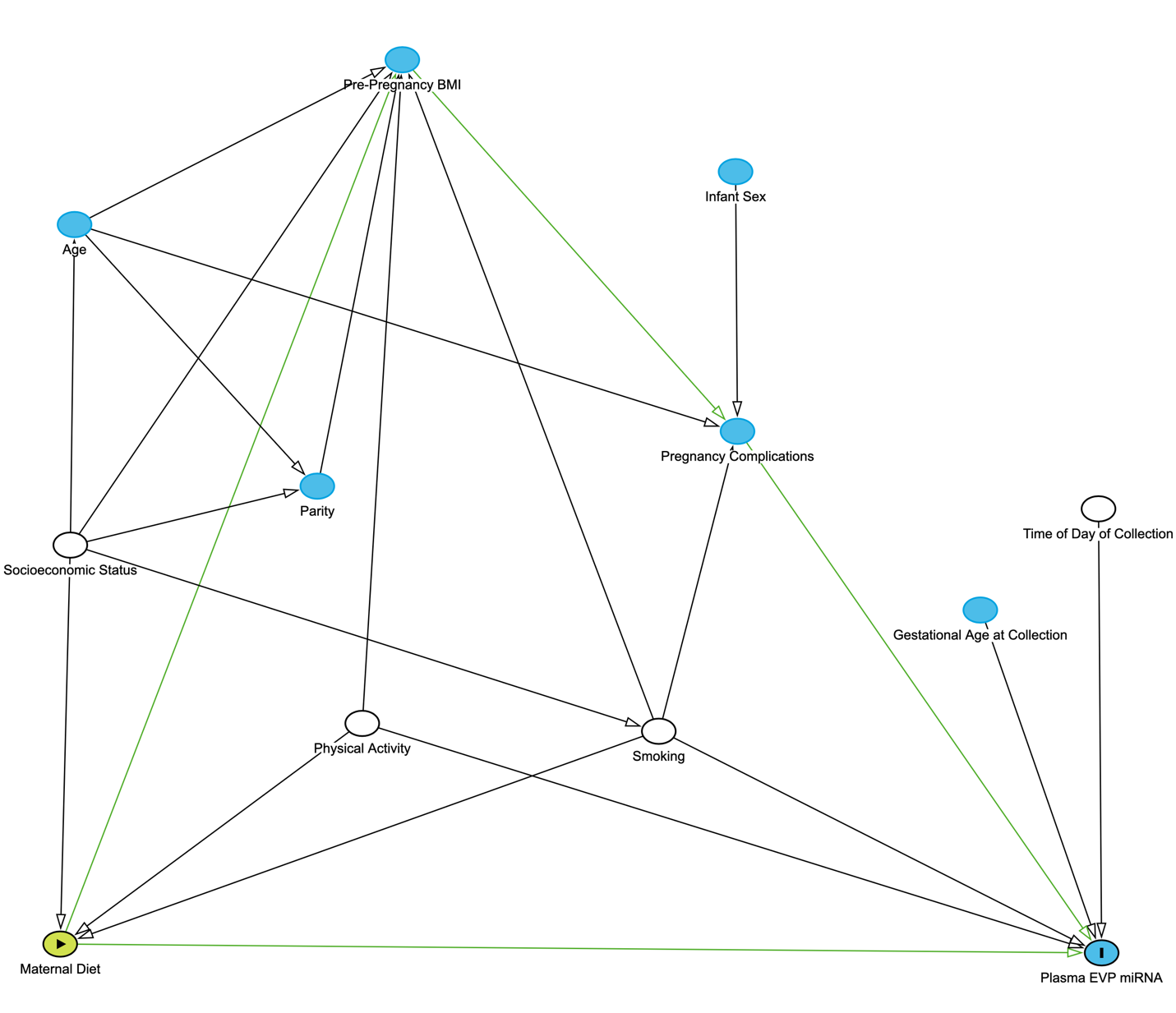
**Figure S1** Directed acyclic graph used to select covariates for models assessing the association between the Alternative Healthy Eating Index 2010 (AHEI-2010; exposure in green) and plasma EVP miRNA composition (outcome in blue). The final adjustment set is highlighted in white. Green arrows reflect causal paths between the exposure and outcome.
